# Preditores de Mortalidade Total e Eventos Arrítmicos Graves em Pacientes com Insuficiência Cardíaca Não Isquêmica: O Papel da Galectina-3

**DOI:** 10.36660/abc.20200353

**Published:** 2021-09-01

**Authors:** Adriano Nunes Kochi, Mauricio Pimentel, Michael Andrades, Tiago Zimerman, Leandro Ioschpe Zimerman, Luis Eduardo Rohde

**Affiliations:** 1 Hospital de Clinicas de Porto Alegre Porto Alegre RS Brasil Hospital de Clinicas de Porto Alegre, Porto Alegre, RS - Brasil; 2 Universidade Federal do Rio Grande do Sul Cardiologia da Faculdade de Medicina Porto Alegre RS Brasil Programa de Pós-Graduação em Ciências Cardiovasculares e Cardiologia da Faculdade de Medicina, Universidade Federal do Rio Grande do Sul, Porto Alegre, RS - Brasil

**Keywords:** Insuficiência Cardíaca, Arritmias Cardíacas, Galectina-3, Apoptose, Desfibriladores Implantáveis, Morte Súbita, Mortalidade

## Abstract

**Fundamento:**

A estratificação de risco continua sendo clinicamente desafiadora em pacientes com insuficiência cardíaca (IC) de etiologia não isquêmica. A galectina-3 é um marcador sérico de fibrose que pode ajudar no prognóstico.

**Objetivo:**

Determinar o papel da galectina-3 como preditora de eventos arrítmicos graves e mortalidade total.

**Métodos:**

Este é um estudo de coorte prospectivo que incluiu 148 pacientes com IC não isquêmica. Todos os pacientes foram submetidos a uma avaliação clínica e laboratorial abrangente para coleta de dados de referência, incluindo níveis de galectina-3 sérica. O desfecho primário foi a ocorrência de síncope arrítmica, intervenções apropriadas do cardioversor desfibrilador implantável, taquicardia ventricular sustentada ou morte súbita cardíaca. O desfecho secundário foi a morte por todas as causas. Para todos os testes estatísticos, considerou-se significativo o valor p<0,05 (bicaudal).

**Resultados:**

Em seguimento mediano de 941 dias, os desfechos primário e secundário ocorreram em 26 (17,5%) e 30 (20%) pacientes, respectivamente. A galectina-3 sérica>22,5 ng/mL (quartil mais alto) não foi preditora de eventos arrítmicos graves (HR: 1,98; p=0,152). Os preditores independentes do desfecho primário foram diâmetro diastólico final do ventrículo esquerdo (DDFVE)>73 mm (HR: 3,70; p=0,001), ventilação periódica durante o exercício (VPE) no teste de esforço cardiopulmonar (HR: 2,67; p=0,01) e taquicardia ventricular não sustentada (TVNS)>8 batimentos na monitorização por Holter (HR: 3,47; p=0,027). Os preditores de morte por todas as causas foram: galectina-3>22,5 ng/mL (HR: 3,69; p=0,001), DDFVE>73 mm (HR: 3,35; p=0,003), VPE (HR: 3,06; p=0,006) e TVNS>8 batimentos (HR: 3,95; p=0,007). A ausência de todos os preditores de risco foi associada a um valor preditivo negativo de 91,1% para o desfecho primário e 96,6% para a mortalidade total.

**Conclusões:**

Em pacientes com IC não isquêmica, níveis elevados de galectina-3 não foram preditores de eventos arrítmicos graves, mas foram associados à mortalidade total. A ausência de preditores de risco revelou um subgrupo prevalente de pacientes com IC com excelente prognóstico.

## Introdução

Apesar dos impressionantes avanços terapêuticos, a insuficiência cardíaca (IC) está associada a taxas de mortalidade persistentemente elevadas^1^ e cerca de 30% da mortalidade total em pacientes com IC é atribuída à morte súbita cardíaca (MSC).^2^^,^^3^ Os cardioversores desfibriladores implantáveis (CDI) são uma estratégia de tratamento estabelecida para prevenir a MSC, particularmente na IC por doença arterial coronariana, já que vários ensaios clínicos demonstraram seus inquestionáveis efeitos benéficos nos desfechos clínicos.^4^^–^^6^ No entanto, há um debate contínuo sobre a eficácia global do implante de CDI em pacientes com cardiomiopatia não isquêmica (CMNI). Estudos clínicos que investigaram esse cenário específico apresentaram resultados conflitantes. Nos ensaios DEFINITE e DANISH, que incluíram apenas pacientes com CMNI, não houve redução da mortalidade por todas as causas, embora tenha sido observada diminuição da MSC.^7^^,^^8^

Essa heterogeneidade na eficácia pode ser parcialmente explicada pela disparidade no risco de MSC para diferentes etiologias de IC. A cardiopatia isquêmica está associada, em particular, a um risco elevado de arritmias potencialmente fatais, atribuíveis, em grande parte, à presença de cicatrizes fibróticas envolvidas na arritmogênese.^6^ A fibrose miocárdica, entretanto, é ubíqua na IC, independentemente da etiologia, e tem sido consistentemente associada ao risco de MSC.^9^^–^^12^ A ressonância magnética cardíaca (RMC) com realce tardio do gadolínio (RTG) é uma ferramenta diagnóstica não invasiva que identifica a fibrose miocárdica e tem sido indicada como potencial preditora de eventos arrítmicos e mortalidade na CMNI.^12^^,^^13^ O uso clínico da RMC-RTG é limitado por seus custos, contraindicações em cenários comuns de IC e disponibilidade restrita em todo o mundo. Um biomarcador sérico simples capaz de identificar a carga da fibrose miocárdica poderia potencialmente ajudar a estratificar o risco de MSC, com uma aplicação mais ampla.^14^^,^^15^ A galectina-3 foi recentemente avaliada como biomarcador de remodelamento cardíaco e fibrose, já que sua produção está diretamente envolvida no início e na evolução da cicatriz tecidual.^15^ Estudos preliminares demonstraram que altos níveis séricos de galectina-3 são preditores de arritmias ventriculares sustentadas em pacientes com IC que tenham alto risco para MSC e eventos cardíacos graves na cardiomiopatia dilatada e hipertrófica.^10^^,^^16^

O objetivo do presente estudo foi avaliar se os níveis de galectina-3 são preditores de eventos arrítmicos e mortalidade total em uma coorte de pacientes com CMNI, agregando assim valor preditivo além dos de outros marcadores de risco conhecidos.^17^

## Métodos

Foi realizado um estudo observacional prospectivo que incluiu pacientes adultos sob tratamento otimizado da IC em um ambulatório dedicado à IC do Hospital de Clínicas de Porto Alegre (Porto Alegre, RS), de março de 2011 a novembro de 2017. Os participantes tinham um diagnóstico prévio de IC sistólica de etiologia não isquêmica. A IC sistólica foi definida como FEVE<40%. Embora os critérios de inclusão possibilitassem a avaliação da FEVE por ecocardiograma transtorácico bidimensional ou RMC, todos os pacientes participantes deste protocolo foram inicialmente examinados por um ecocardiograma, de preferência pelo método biplanar de Simpson. A etiologia não isquêmica foi definida como ausência de lesões coronarianas ateroscleróticas>75% na angiografia coronariana ou ausência de áreas necróticas ou isquêmicas na tomografia computadorizada por emissão de fóton único (*single-photon emission computed tomography* — SPECT) ou na RMC-RTG. Os critérios de exclusão foram histórico de MSC, síncope cardiogênica prévia, taquicardia ventricular (TV) sustentada prévia, doença cerebrovascular avançada ou expectativa de vida inferior a um ano por doenças não cardiovasculares. O protocolo do estudo foi aprovado pelo Comitê de Ética do Hospital de Clínicas de Porto Alegre e o termo de consentimento livre e esclarecido foi obtido de todos os participantes. Os procedimentos do protocolo de estudo envolveram uma avaliação clínica detalhada, exames laboratoriais de rotina, incluindo peptídeo natriurético tipo B (BNP) ou N-terminal do pró-peptídeo natriurético tipo B (NT-proBNP), exames cardíacos não invasivos e estudo eletrofisiológico (EEF) invasivo. Os exames cardíacos não invasivos consistiram em eletrocardiograma de repouso (ECG) de rotina, gravação contínua 24 h do ECG (Holter) e teste de esforço cardiopulmonar (TECP). Durante o EEF invasivo, o sangue foi coletado para a análise da galectina-3.

### ECG e monitorização por Holter de 24 h

O ECG de 12 derivações foi realizado com um aparelho digital (Mortara ELI 350, Mortara Instrument, Milwaukee, Wisconsin, EUA) e a monitorização por Holter de 24 h foi feita com um gravador digital (SEER Light) e analisado com o programa GE Marquette MARS (GE Healthcare, Wauwatosa, Wisconsin, EUA) por um cardiologista experiente. A TV não sustentada (TVNS) foi definida como uma série de 3 ou mais batimentos ventriculares prematuros consecutivos≥100 bpm.

### Teste de esforço cardiopulmonar (TECP)

O TECP foi realizado em esteira padrão (INBRAMED^TM^ KT 10200, Porto Alegre, Brasil) com um analisador de gás computadorizado calibrado (Cortex Biophysik Metalyzer 3B Stationary CPX system, M13B2.1, Leipzig, Alemanha). Foi utilizado um protocolo de rampa por etapas, começando a 2,4 km/h com inclinação de 1–2%, seguido de incrementos progressivos de velocidade de 0,1–0,12 km/h a cada 20 s e incrementos de inclinação de 0,5–1,0% a cada 60 s, até atingir a fadiga volicional. As variáveis tradicionais do TECP foram avaliadas: consumo máximo de oxigênio (pico de VO_2_), produção de dióxido de carbono (VCO_2_), ventilação minuto (VE), razão de troca respiratória (RTR), inclinação VE/VCO_2_ e ventilação periódica durante o exercício (VPE). A VPE foi definida com base nos seguintes critérios: (1) 3 ou mais oscilações regulares, claramente discerníveis do ruído de fundo, (2) regularidade, definida como desvio padrão (DP) de 3 ciclos consecutivos (tempo entre 2 nadires consecutivos) dentro de 20% da média e (3) amplitude média mínima de oscilação ventilatória de 5 L (valor do pico menos a média entre 2 nadires intermediários consecutivos).

### Estudo eletrofisiológico invasivo

Os pacientes foram sedados com midazolam e fentanil e anestesiados localmente com lidocaína. Um cateter diagnóstico quadripolar foi introduzido pela veia femoral direita e posicionado sob fluoroscopia no ápice ventricular direito. O sistema EP-Tracer (CardioTek^TM^, Maastricht, Países Baixos) foi utilizado para a estimulação ventricular programada (EVP), com uma amplitude do pulso de saída de duas vezes o limite e largura de pulso de 1 ms. O protocolo de estimulação consistiu em até 3 extra-estímulos (S2/S3/S4), administrados após ciclos de 10 batimentos. Foram realizados decrementos de 10 milissegundos no intervalo de acoplamento do extra-estímulo após cada ciclo, até que a refratariedade ventricular fosse alcançada ou o intervalo de acoplamento atingisse 200 ms. Esse processo foi repetido em 3 ciclos básicos (600, 500 e 400 ms). A TV sustentada foi definida como taquicardia monomórfica ou polimórfica com duração de ≥30 s ou colapso hemodinâmico. Quando nenhuma TV sustentada foi induzida, a EVP foi repetida com até 2 extra-estímulos (S2/S3) após infusão venosa de isoproterenol (1–4 mcg/min). A indução de TV monomórfica ou polimórfica ou de fibrilação ventricular (FV) com extra-estímulos triplos foi considerada um achado verdadeiramente positivo na análise atual. Em pacientes com marcapasso permanente implantado anteriormente, um EEF não invasivo foi realizado com o programador de marcapasso, sob o mesmo protocolo.

### Medição da galectina-3 sérica

Uma amostra de 20 mL de sangue foi coletada por meio da bainha venosa antes do EEF invasivo. A amostra de sangue foi centrifugada em um laboratório de pesquisa dedicado e armazenada a -70ºC. A galectina-3 foi medida duas vezes, utilizando o teste ELISA (BG Medicine, Waltham, EUA).

### Medição de peptídeos natriuréticos

No ambulatório de IC onde os pacientes foram acompanhados, tanto os níveis de BNP como os de NT-proBNP estavam disponíveis durante o período do estudo. Para esta análise, pacientes com níveis elevados de peptídeos natriuréticos foram definidos como aqueles que estavam no quartil mais alto de qualquer um deles.

### Seguimento e desfechos

Os pacientes tiveram consultas ambulatoriais aos 3, 6, 18, 24, 30 e 36 meses. Aqueles que não compareceram às consultas de seguimento foram contatados por telefone ou receberam visitas domiciliares. A decisão de implantar o CDI foi tomada pela equipe clínica de cardiologia envolvida no atendimento de rotina, sem interferência dos pesquisadores. O desfecho primário do protocolo consistiu em eventos arrítmicos (MSC, TV sustentada, síncope cardíaca ou intervenção apropriada do CDI). Choques do CDI foram considerados adequados se causados por TV ou FV. Não havia protocolo padronizado de programação do CDI para o tratamento de taquicardia, mas a zona de FV foi tipicamente definida como >200 bpm com pelo menos 1 episódio de estimulação antitaquicardia (*antitachycardia pacing* — ATP) antes do choque, enquanto a zona de TV foi tipicamente definida como >180 bpm com pelo menos 3 episódios de ATP antes do choque. O desfecho secundário do estudo foi a morte por todas as causas. Os desfechos foram adjudicados por dois pesquisadores independentes e cegos para a avaliação inicial. Casos discordantes foram definidos por consenso.

### Análises estatísticas

Os dados são expressos como média±DP ou mediana e intervalo interquartil (IIQ) para as variáveis contínuas de acordo com a normalidade dos dados ou como números absolutos e porcentagem para as variáveis categóricas. Dados contínuos foram adicionados ao modelo de regressão e categorizados com base em quartis de distribuição em 2 grupos: pacientes com valores abaixo do percentil 75 (<quartil 3 [Q_3_]) e pacientes com valores iguais ou superiores ao percentil 75(≥Q_3_). Para determinar a normalidade de todas as variáveis contínuas, foi utilizado o teste de Shapiro-Wilk. As comparações entre os grupos foram realizadas usando o teste *t* de Student não pareado para variáveis contínuas e o teste qui-quadrado ou o teste exato de Fischer para variáveis categóricas. Variáveis sem distribuição normal foram comparadas pelo teste U de Mann-Whitney. Para os valores ausentes do TECP (n=13), os dados foram imputados de acordo com cinco modelos multivariados, construídos a partir de variáveis capazes de predizer a VPE, com base nas estratégias de imputação previamente validadas de Rubin,^18^ que fornecem valores de entrada sem perder a precisão dos dados. A taxa de sobrevida e a sobrevida livre de eventos arrítmicos graves nos dois grupos foram determinadas pelo método de Kaplan-Meier e a diferença entre eles foi analisada pelo teste log-rank. A regressão de Cox foi adotada para análises univariadas e multivariadas de potenciais preditores dos desfechos primário e secundário. Valores p bicaudais iguais ou inferiores a 0,05 foram considerados estatisticamente significativos. Com base na incidência de 26% de terapias com CDI de um estudo de coorte composto por CMNI e IC isquêmica, foi calculado um tamanho amostral de 142 pacientes (80% de poder estatístico e valor p bicaudal<0,05). Todas as análises foram realizadas utilizando o software estatístico SPSS (versão 19; Chicago, EUA).

## Resultados

### Características dos pacientes

Foram incluídos 148 dos 296 pacientes ambulatoriais com IC avaliados. A maioria era do sexo masculino (59,5%), com idade média de 54,8±13 anos. A etiologia da IC foi cardiomiopatia idiopática em 45,4% dos casos, cardiomiopatia hipertensiva em 16,9% e cardiomiopatia alcoólica em 12,2%. A maioria dos participantes apresentava classe funcional da New York Heart Association (NYHA) I ou II (42,6% e 39,9%, respectivamente) e a FEVE média foi de 27,4±7,5%. O tratamento farmacológico foi otimizado na maioria dos pacientes: 97% faziam uso de um betabloqueador, juntamente com um inibidor da enzima conversora da angiotensina ou um bloqueador do receptor da angiotensina, 70% estavam tomando espironolactona e 81% estavam usando digoxina. A Tabela 1 descreve as características clínicas de toda a coorte estratificadas pelos níveis de galectina-3.

**Tabela 1:** Características clínicas de acordo com os níveis de galectina-3

		Todos os pacientes (n=148)	Quartil superior GAL-3>22,5 ng/mL (n=36)	Quartis inferiores GAL-3≤22,5 ng/mL (n=112)	Valor p
Idade (anos)		54,8±12,7	63±9,3	52,2±12,6	<0,001
Sexo masculino		88 (59,5)	20 (55,6)	68 (61,3)	0,54
**Classe NYHA (%)**					
	I	63 (42,6)	11 (30,6)	51 (46)	0,12
	II	59 (39,9)	15 (41,7)	44 (40)	
	III	26 (17,6)	10 (28,8)	16 (14,4)	
	IV	0	0 (0)	0 (0)	
**Etiologia (%)**					
	Idiopática	67 (45,3)	13 (36,1)	54 (48,6)	0,38
	Hipertensiva	25 (16,9)	7 (19,4)	18 (16,2)	
	Alcoólica	18 (12,2)	4 (11,1)	14 (12,6)	
	Doença de Chagas	7 (4,7)	3 (8,3)	4 (3,6)	
	Valvar	4 (2,7)	2 (5,6)	2 (1,8)	
	Outra	27 (18,2)	7 (19,5)	19 (17,1)	
**Exame físico**					
PAS (mmHg)		119,3±21,6	122,6±22,5	118,1±21,3	0,28
PAD (mmHg)		74,7±12,7	74,6±13	74,8±12,8	0,95
**Exames laboratoriais**					
Hemoglobina (g/dL)		13,4±1,6	12,6±1,9	13,6±1,4	0,008
Linfócitos (/mm³)		2099,2±848	1825±787	2193±852	0,02
Creatinina (mg/dL)		1,1±0,73	1,6±1,1	1,0±0,4	0,002
Sódio (mEq/L)		140±2,8	140±2,9	140±2,8	0,86
Potássio (mEq/L)		4,6±0,4	4,6±0,4	4,6±0,3	0,82
Ácido úrico (mg/dL)		7,5±2,2	8,6±2,4	7,1±2,1	<0,001
Glicose (mg/dL)		118±49,6	117,5±40,3	118,4±52,6	0,92
Colesterol total (mg/dL)		180,6±42,6	188,4±42,6	177,3±41	0,16
LDL (mg/dL)		104,7±37,2	110,5±37,6	102,3±36,8	0,26
Galectina-3 (ng/mL)		19±9,4	31,6±10,7	14,9±10,7	<0,001
BNP (pg/mL)		116,4 (59,7–295)	158 (77–289)	106,6 (53–298)	0,30
NT-proBNP (pg/mL)		1145 (392–2590)	4776 (1549–15852)	741 (314–2291)	0,005
**Ecocardiograma**					
	FEVE (%)	27,4±7,5	27,3±7,6	27,4±7,5	0,97
	Átrio esquerdo (mm)	47,3±6,6	48,7±7	46,9±6,4	0,15
	DDFVE (mm)	67,5±10,2	65,3±7	68,2±11	0,13
	DSFVE (mm)	58,7±10,1	56,6±8,5	59,4±10,6	0,15
**ECG**					
Fibrilação atrial		22 (14,9)	8 (22)	14 (12,6)	0,16
BRE		60 (40,8)	18 (50)	42 (37,8)	0,46
**Monitorização por Holter de 24 horas**					
TVNS (%)		54 (36,5)	12 (33,3)	42 (38,5)	0,57
TVNS>8 batimentos		11 (7,4)	1 (8,3)	9 (21,4)	0,54
**Teste de esforço cardiopulmonar**					
VO_2_ máximo (mL/kg/min)		18±5,1	14,7±4,3	19±4,9	<0,001
Inclinação VE/VCO_2_		41,5±11,7	44,3±12,5	40,8±11,5	0,14
VPE (%)		26 (17,5)	3 (8,3)	23 (20,5)	0,14
**EEF (%)**					
Sem indução		129 (87,2)	34 (94,4)	94 (84,7)	0,10
TVMS		10 (6,8)	0	10 (9)	
TVPS		5 (3,4)	1 (2,8)	4 (3,6)	
Fibrilação ventricular		3 (2)	0	3 (2,7)	
Intervalo HV (ms)		52,6±10,4	54,2±11,3	52,2±10,1	0,34
**Medicação**					
Betabloqueador (%)		144 (97,3)	36 (100)	107 (96,4)	0,57
IECA ou BRA (%)		144 (97,3)	32 (88,9)	111 (99,1)	0,003
Espironolactona (%)		103 (69,6)	21 (58,3)	82 (73,9)	0,07
Digoxina (%)		121 (81,8)	31 (86,1)	90 (81,1)	0,49
Medicamento antiarrítmico (%)		8 (5,4)	1 (2,8)	7 (6,3)	0,41

Os dados foram expressos como média±desvio padrão, mediana (Q1–Q3) ou números absolutos (porcentagem). GAL-3: galectina-3; NYHA: New York Heart Association; PAS: pressão arterial sistólica; PAD: pressão arterial diastólica; LDL: lipoproteína de baixa densidade; BNP: peptídeo natriurético tipo B; NT-proBNP: N-terminal do pró-peptídeo natriurético tipo B; FEVE: fração de ejeção do ventrículo esquerdo; DDFVE: diâmetro diastólico final do ventrículo esquerdo; DSFVE: diâmetro sistólico final do ventrículo esquerdo; ECG: eletrocardiograma; TVNS: taquicardia ventricular não sustentada; VO_2_: consumo de oxigênio; inclinação VE/VCO_2_: eficiência ventilatória; VPE: ventilação periódica durante o exercício; EEF: estudo eletrofisiológico; TVMS: taquicardia ventricular monomórfica sustentada; TVPS: taquicardia ventricular polimórfica sustentada; IECA: inibidor da enzima conversora da angiotensina; BRA: bloqueador do receptor de angiotensina II; BRE: bloqueador do ramo esquerdo.

### Seguimento e desfechos

O seguimento mediano foi de 941 dias (IIQ: 440–1241 dias). Todos os pacientes tiveram pelo menos duas consultas de seguimento e apenas três não puderam ser encontrados. O desfecho primário (Tabela 2) ocorreu em 26 pacientes (17,5%): MSC em 13 (8,7%), síncope cardíaca em 5 (3,3%), intervenção apropriada do CDI em 7 (4,7%) e TV sustentada em 1 (0,7%). Durante o seguimento, 30 (20,2%) pacientes morreram e a 10 deles foi atribuída morte cardiovascular (6,7%). Oito pacientes (5,4%) foram submetidos a transplante cardíaco. Houve 81 internações por IC descompensada. Quarenta e oito pacientes foram submetidos a implante de dispositivo: 17 terapias de ressincronização cardíaca associadas ao CDI (TRC-D), 19 CDI de câmara única, 5 terapias de ressincronização cardíaca associadas ao marcapasso (TRC-M), 4 CDI de dupla câmara e 4 marcapassos de câmara única.

**Tabela 2:** Análise univariada e modelo de risco proporcional de Cox para o desfecho primário (eventos arrítmicos graves)

	Análise univariada			Análise multivariada		
	HR	IC95%	p	HR	IC95%	p
Galectina-3 (para cada 1 ng/mL)	1,003	0,97–1,04	0,877			
Galectina-3>22,5 ng/mL	1,13	0,47–2,70	0,787			
Fibrilação atrial	1,86	0,75–4,60	0,182			
FEVE<20%	0,69	0,21–2,30	0,691			
DDFVE>73 mm	4,13	1,91–8,90	<0,001	3,70	1,69–8,1	0,001
VO_2_ máximo<14,2 mL/kg/min	1,69	0,75–3,90	0,203			
Inclinação VE/VCO_2_>48,4	2,32	1,05–5,10	0,037			
VPE	3,37	1,52–7,40	0,030	2,67	1,19–6,0	0,017
Intervalo HV>59 ms	2,23	1,01–4,90	0,047			
TVNS>8 batimentos	3,27	1,11–9,70	0,030	3,47	1,15–10,5	0,027
EEF positivo	1,58	0,54–4,60	0,403			
Peptídeos natriuréticos elevados	2,75	1,26–6,01	0,011			

HR (*hazard ratio*): razão de risco; FEVE: fração de ejeção do ventrículo esquerdo; DDFVE: diâmetro diastólico final do ventrículo esquerdo; VO_2_ máximo: consumo máximo de oxigênio; TVNS: taquicardia ventricular não sustentada; VPE: ventilação periódica durante o exercício; EEF: estudo eletrofisiológico invasivo.

### Níveis de galectina-3

O nível médio de galectina-3 foi de 19±9,4 ng/mL e a mediana foi de 16 ng/mL (IIQ: 13,1–22,5). Os dados clínicos estratificados pelo quartil superior dos níveis de galectina-3 (>22,5 ng/mL) são apresentados na Tabela 1. Os níveis de galectina-3 não diferiram nos pacientes com ou sem o desfecho primário e os quartis de galectina-3 (>22,5 ng/mL) também não diferenciaram pacientes com eventos arrítmicos graves (7 [19,4%] pacientes no quartil superior versus 19 [17%] pacientes nos quartis inferiores; p=0,73). Entretanto, os níveis de galectina-3 estratificados por quartis foram significativamente diferentes para a mortalidade total (13 [36,1%] pacientes no quartil superior versus 17 [15,2%] pacientes nos quartis inferiores; p=0,007) e para hospitalização por IC (50 [34%] pacientes no quartil superior versus 31 [21%] pacientes nos quartis inferiores; p<0,001) durante o seguimento.

### Análises univariadas e multivariadas

Em análises univariadas, os preditores significativos de eventos arrítmicos graves (Tabela 2) foram o quartil mais alto do diâmetro diastólico final do ventrículo esquerdo (DDFVE) ao ecocardiograma (razão de risco [*hazard ratio* — HR]: 4,13; p<0,001), o quartil mais alto da inclinação VE/VCO_2_ (HR: 2,32; p=0,03), a presença de VPE no TECP (HR: 3,37; p=0,03), o quartil mais alto do intervalo HV no EEF invasivo (HR: 2,23; p=0,04) e a TVNS>8 batimentos na monitorização por Holter (HR: 3,27; p=0,03). Nesta análise, os níveis de galectina-3, tanto os contínuos como os estratificados por quartis, não foram preditores significativos do desfecho primário (HR: 1,13; p=0,78). No modelo multivariado de regressão de Cox, as variáveis que permaneceram significativamente associadas a eventos arrítmicos graves foram DDFVE (HR: 3,70; p=0,001), presença de VPE (HR: 2,67; p=0,01) e TVNS>8 batimentos (HR: 3,47; p=0,027). Resultados semelhantes foram obtidos utilizando o DDFVE indexado para a superfície corpórea (DDFVE>40 mm/m^2^ representando o percentil 75; HR: 3,34; intervalo de confiança de 95% 1,50–7,45; p=0,003).

Os preditores de mortalidade total em análises univariadas foram semelhantes, mas também incluíram o quartil mais alto dos níveis de galectina-3 (HR: 2,20; p=0,03), o VO_2_ máximo (HR: 0,92; p=0,04) e o quartil mais alto do intervalo HV (HR: 2,80; p=0,005). Os preditores independentes de mortalidade total no modelo multivariado (Tabela 3) foram o maior quartil de galectina-3 (HR: 3,69; p=0,001), o quartil mais alto do DDFVE (HR: 3,35; p=0,003), a presença de VPE (HR: 3,06; p=0,006) e a TVNS>8 batimentos (HR: 3,95; p=0,007). Resultados semelhantes foram obtidos utilizando o DDFVE indexado para a superfície corpórea (DDFVE>40 mm/m^2^ representando o percentil 75; HR: 3,77; intervalo de confiança de 95% 1,77–8,02; p=0,001).

**Tabela 3:** Análise univariada e modelo de risco proporcional de Cox para o desfecho secundário (mortalidade total)

	Análise univariada			Análise multivariada		
	HR	IC95%	p	HR	IC95%	p
Galectina-3 (para cada 1 ng/mL)	1,024	0,99–1,05	0,057			
Galectina-3>22,5 ng/mL	2,20	1,07–4,50	0,033	3,69	1,7–8,19	0,001
Fibrilação atrial	0,98	0,34–2,80	0,980			
FEVE (para cada 1%)	0,96	0,91–1,01	0,098			
FEVE<20%	1,40	0,57–3,40	0,461			
DDFVE>73 mm	3,02	1,44–6,30	0,003	3,35	1,53–7,34	0,003
VO_2_ máximo (mL/kg/min)	0,92	0,86–0,99	0,042			
VO_2_ máximo<14,2 mL/kg/min	1,20	0,53–2,70	0,656			
Inclinação VE/VCO_2_>48,4	1,92	0,89–4,10	0,092			
VPE	2,91	1,36–6,20	0,006	3,06	1,38–6,77	0,006
Intervalo HV>59 ms	2,80	1,36–5,80	0,005	1,98	0,95–4,13	0,068
TVNS>8 batimentos	3,31	1,26–8,70	0,015	3,95	1,45–10,73	0,007
EEF positivo	0,95	0,29–3,10	0,993			
Peptídeos natriuréticos elevados	3,44	1,67–7,06	0,001			

HR (*hazard ratio*): razão de risco; FEVE: fração de ejeção do ventrículo esquerdo; DDFVE: diâmetro diastólico final do ventrículo esquerdo; VO_2_ máximo: consumo máximo de oxigênio; VPE: ventilação periódica durante o exercício; TVNS: taquicardia ventricular não sustentada; EEF: estudo eletrofisiológico invasivo.

### Valores preditivos

Os valores preditivos positivos (VPP) para o desfecho primário foram baixos para os parâmetros individuais (Tabela 4); a única variável associada ao maior VPP foi a VPE (38,4%), enquanto a variável com maior valor preditivo negativo (VPN) foi o DDFVE>73 mm (88,3%). Noventa pacientes (61% da amostra estudada) não apresentaram nenhuma das 3 variáveis independentemente associadas ao desfecho primário, levando a um VPN de 91,1%. Achados semelhantes foram observados para mortalidade total: TVNS>8 batimentos foi o preditor associado ao maior VPP (45,5%), enquanto o intervalo HV>59 ms obteve o maior VPN (84,9%). Pacientes sem nenhuma das 5 variáveis independentemente associadas ao risco apresentaram VPN de 96,3% para morte por todas as causas.

**Tabela 4:** Valores preditivos positivos e negativos de acordo com fatores de risco

Desfecho primário (eventos arrítmicos graves)				
	Todos os pacientes (n=148)	Com evento (n=26)	VPP% (IC95%)	VPN% (IC95%)
DDFVE>73 mm	36	13	36,1 (24,9–49)	88,3 (83,7–91,8)
VPE	26	10	38,4 (24,2–54,9)	86,8 (82,9–90)
TVNS>8 batimentos	11	4	36,3 (15,2–64,4)	83,7 (81,2–85,9)
Ausência de todos os três	90	8		91,1 (85,4–94,8)
**Desfecho secundário (mortalidade total)**				
	**Todos os pacientes (n=148)**	**Com evento (n=30)**	**VPP% (IC95%)**	**VPN% (IC95%)**
GAL-3>22,5 ng/mL	36	13	36,1 (24,6–49,4)	84,8 (80,1–88,5)
DDFVE>73 mm	36	12	33,3 (22,1–46,8)	83,9 (79,3–87,6)
VPE	26	10	38,4 (24,3–55,2)	83,6 (79,6–86,9)
TVNS>8 batimentos	11	5	45,5 (21,4–71,8)	81,4 (78,8–83,8)
Intervalo HV>59 ms	35	13	37,1 (25,3–50,7)	84,9 (80,3–88,6)
Ausência de todos os cinco	55	2		96,3 (85,2–99,0)

GAL-3: galectina-3; DDFVE: diâmetro diastólico final do ventrículo esquerdo; VPE: ventilação periódica durante o exercício; TVNS: taquicardia ventricular não sustentada.

**Figura 1:**
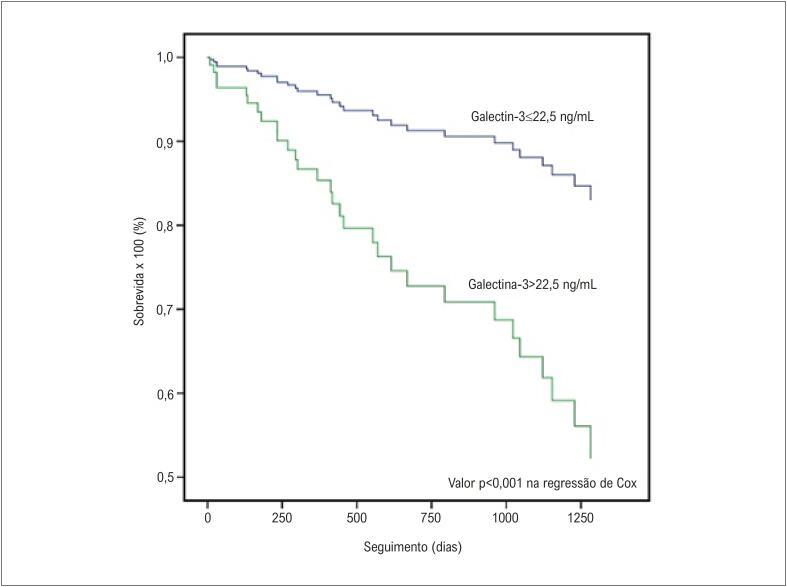
Curvas determinadas por regressão de Cox para a mortalidade total de acordo com níveis séricos de galectina-3. diâmetro diastólico final do ventrículo esquerdo >73mm, ventilação periódica, taquicardia ventricular não-sustentada >8 batimentos e intervalo HV >59ms.

**Figura 2:**
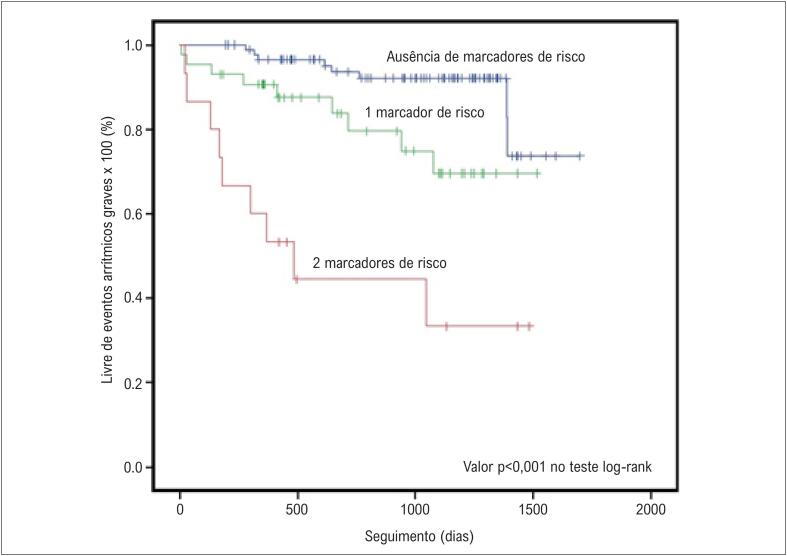
Curva de sobrevida de Kaplan-Meier para o desfecho primário (eventos arrítmicos graves) estratificado pelo número de fatores de risco (diâmetro diastólico final do ventrículo esquerdo dilatado, ventilação periódica durante o exercício no teste de esforço cardiopulmonar e taquicardia ventricular não sustentada na monitorização por Holter).

**Figura 3:**
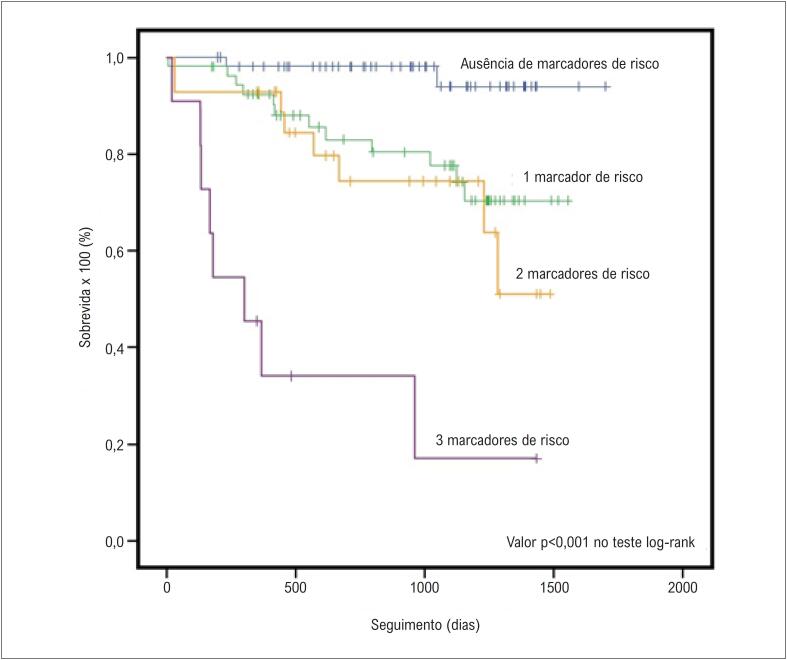
Curva de sobrevida de Kaplan-Meier para o desfecho secundário (mortalidade total) estratificado pelo número de fatores de risco (níveis elevados de galectina-3, diâmetro diastólico final do ventrículo esquerdo dilatado, ventilação periódica durante o exercício no teste de esforço cardiopulmonar, taquicardia ventricular não sustentada na monitorização por Holter e aumento do intervalo HV).

A Figura 1 demonstra as curvas de sobrevida para diferentes níveis de galectina-3, ajustadas para os outros preditores de mortalidade por todas as causas no modelo de regressão de Cox (p<0,001). A Figura 2 mostra a curva de sobrevida de Kaplan-Meier para o desfecho primário estratificado pelo número de variáveis preditoras (DDFVE, VPE, TVNS>8 batimentos; valor p<0,001 no teste log-rank). A Figura 3 apresenta a curva de sobrevida de Kaplan-Meier para a mortalidade por todas as causas estratificada pelo número de marcadores de risco (galectina-3>22,5 ng/mL, DDFVE>73 mm, VPE, TVNS>8 batimentos, intervalo HV>59 ms). Pacientes com IC e mais de 3 fatores de risco tinham um prognóstico alarmante, com taxa de mortalidade>80% após 3 anos de seguimento.

## Discussão

Nesta coorte prospectiva de pacientes com IC-CMNI, os níveis de galectina-3 não foram preditores independentes de eventos arrítmicos graves (MSC, síncope cardíaca, TV sustentada ou intervenção apropriada do CDI). No entanto, níveis mais elevados de galectina-3 foram independentemente associados à mortalidade total. Anteriormente, havíamos identificado 3 preditores clínicos de eventos arrítmicos (DDFVE, VPE e TVNS na monitorização por Holter de 24 horas), os quais foram confirmados pela análise atual.^17^ Em um cenário clínico em que os implantes de CDI estão sob escrutínio, os dados do presente estudo podem ajudar a selecionar os pacientes que mais se beneficiariam com uma terapia invasiva e dispendiosa. Níveis séricos de galectina-3 podem ser utilizados para estratificar ainda mais o risco e ajudar no prognóstico.^18^

As galectinas são uma grande família de lectinas que se ligam a ß-galactosídeos. Localizadas principalmente no citoplasma, podem ser encontradas também no núcleo ou na matriz extracelular. A galectina-3 extracelular apresenta diversos efeitos autócrinos e parácrinos, como adesão celular, ativação e quimioatração de certos tipos de células, principalmente os relacionados à matriz extracelular. A galectina-3 afeta vários processos biológicos, como a homeostase celular, respostas imunes, a organogênese e a angiogênese.^19^ Henderson et al. relataram que a interrupção do gene da galectina-3 bloqueou a ativação das células estreladas hepáticas e a expressão do colágeno no fígado, atenuando a fibrose hepática.^20^ A aldosterona levou à expressão da galectina-3 na túnica média da aorta em modelos animais e, por sua vez, sua superexpressão aumentou a produção de colágeno tipo I.^21^

O papel da galectina-3 como preditora de eventos clínicos futuros tem sido parcialmente avaliado em vários cenários cardiovasculares. Maiores níveis de galectina-3 foram relacionados ao surgimento de fibrilação atrial (FA) 3–5 dias após o infarto do miocárdio com supradesnivelamento do segmento ST.^22^ Relatos recentes também indicaram associação de níveis de galectina-3 com a FA paroxística^23^ e a FA persistente.^24^ As concentrações de sST2 e galectina-3 foram significativamente maiores em pacientes com cardiomiopatia hipertrófica do que nos controles, mas nenhum dos marcadores apresentou relação significativa com o risco de MSC, histórico de síncope ou histórico familiar de MSC.^25^

O papel preditivo da galectina-3 também tem sido avaliado em estudos de etiologias mistas de IC, mas poucos têm explorado seu papel como preditora de eventos arrítmicos especificamente em pacientes com IC não isquêmica. Francia et al.,^16^ investigaram 75 pacientes com IC submetidos ao implante de CDI e identificaram que os níveis de galectina-3 foram maiores naqueles com TV ou FV. A maior parte daquela amostra (60%), entretanto, tinha etiologia isquêmica.^16^ Hu et al.,^10^ avaliaram os níveis de galectina-3 e o RTG na RMC em uma coorte prospectiva que incluiu pacientes com CMNI (46% com cardiomiopatia dilatada e 56% com cardiomiopatia hipertrófica). Tanto os níveis de galectina-3 como a presença de RTG foram preditores independentes de eventos cardíacos graves. Não foi realizada análise específica relacionada ao risco arrítmico ou à mortalidade total.^10^ Recentemente, pesquisadores da Competence Network Heart Failure avaliaram o papel do sST2 e da galectina-3 em pacientes com cardiomiopatia dilatada não isquêmica e chegaram a resultados intrigantes. Enquanto o sST2 foi associado à mortalidade cardíaca e total, os níveis de galectina-3 não tiveram impacto no risco de eventos futuros como variável contínua, mas o tercil intermediário da galectina-3 foi significativamente associado a um melhor prognóstico.^26^ No entanto, os achados do presente estudo sugerem que níveis elevados de galectina-3 podem não ser preditores de risco arrítmico em uma amostra selecionada de pacientes com IC não isquêmica, mas podem ser um marcador importante de mortalidade total. Esses resultados estão em conformidade com uma meta-análise de 9 estudos que incluiu um grupo heterogêneo de pacientes com IC e revelou que, para cada 1 ng/mL de galectina-3, a taxa de mortalidade aumentava em 28%.^27^

Na análise atual, a ausência de todos os preditores independentes de risco para eventos arrítmicos (Figura 2) ou mortalidade total (Figura 3) revelou um subgrupo de pacientes com excelente prognóstico em um seguimento de até quatro anos. Estes resultados reforçam nossos achados prévios^17^ e indicam que uma melhor estratificação de risco é viável em pacientes com IC não isquêmica.

Alguns aspectos metodológicos deste protocolo merecem consideração. Perdeu-se contato com apenas três pacientes, por isso eles foram censurados na última consulta. Arritmias ventriculares assintomáticas não puderam ser detectadas, pois somente 48 pacientes desta coorte foram submetidos a implante de dispositivo. A RMC-RTG vem sendo sugerida como uma ferramenta válida para a estratificação de prognóstico da IC, mas apenas alguns poucos pacientes desta pesquisa foram submetidos à RMC, impedindo sua análise como fator prognóstico nesta coorte. A RMC, contudo, não está amplamente disponível para a maioria dos pacientes com IC em todo o mundo. Sabe-se que o tamanho desta amostra e o número de eventos são relativamente pequenos e, dessa forma, os resultados apresentados devem ser considerados como geradores de hipóteses. Ainda assim, a maioria dos estudos anteriores tinha tamanhos de amostra semelhantes ou menores. Por fim, os achados deste estudo merecem futura validação prospectiva antes que uma ampla aplicabilidade clínica possa ser proposta.

## Conclusão

Nesta coorte prospectiva de pacientes com IC não isquêmica sob tratamento médico otimizado, os níveis de galectina-3 não foram preditores de eventos arrítmicos graves. Três variáveis foram confirmadas como marcadores de risco para o desfecho primário: DDFVE>73 mm, VPE no TECP e TVNS>8 batimentos na monitorização por Holter. Níveis elevados de galectina-3 foram independentemente associados à mortalidade total. A ausência de todos os preditores de risco revelou um subgrupo significativamente prevalente de pacientes com excelente prognóstico. No cenário atual de incertezas sobre as vantagens do CDI em pacientes com IC-CMNI, estes resultados podem ajudar a determinar estratégias futuras para identificar os pacientes que mais se beneficiariam com o CDI.
