# Supplementary material for: Preditores de Mortalidade Total e Eventos Arrítmicos Graves em Pacientes com Insuficiência Cardíaca Não Isquêmica: O Papel da Galectina-3
Source: Arq Bras Cardiol. 2021 Sep 1;117(3):531–41. [Article in Portuguese] doi: 10.36660/abc.20200353 (PMC8462942; doi:10.36660/abc.20200353)
Supplement: Translation

# Translation: Predictors of Total Mortality and Serious Arrhythmic Events in Non-Ischemic Heart Failure Patients: The Role of Galectin-3

**Authors:** Adriano Nunes Kochi, Mauricio Pimentel, Michael Andrades, Tiago Zimerman, Leandro Ioschpe Zimerman, Luis Eduardo Rohde

**Affiliations:** 1 Porto Alegre RS Brazil Hospital de Clinicas de Porto Alegre, Porto Alegre, RS - Brazil; 2 Porto Alegre RS Brazil Programa de Pós-Graduação em Ciências Cardiovasculares e Cardiologia da Faculdade de Medicina, Universidade Federal do Rio Grande do Sul, Porto Alegre, RS - Brazil

**Keywords:** Heart Failure, Arrhythmias Cardiacs, Galectin-3, Apoptosis, Defibrillators, Implantable, Death Sudden, Mortality

## Abstract

**Background:**

Risk stratification remains clinically challenging in patients with heart failure (HF) of non-ischemic etiology. Galectin-3 is a serum marker of fibrosis that might help in prognostication.

**Objective:**

To determine the role of galectin-3 as a predictor of major arrhythmic events and overall mortality.

**Methods:**

We conducted a prospective cohort study that enrolled 148 non-ischemic HF patients. All patients underwent a comprehensive baseline clinical and laboratory assessment, including levels of serum galectin-3. The primary outcome was the occurrence of arrhythmic syncope, appropriate implantable cardioverter defibrillator therapy, sustained ventricular tachycardia, or sudden cardiac death. The secondary outcome was all-cause death. For all statistical tests, a two-tailed p-value<0.05 was considered significant.

**Results:**

In a median follow-up of 941 days, the primary and secondary outcomes occurred in 26 (17.5%) and 30 (20%) patients, respectively. Serum galectin-3>22.5 ng/mL (highest quartile) did not predict serious arrhythmic events (HR: 1.98, p=0.152). Independent predictors of the primary outcome were left ventricular end-diastolic diameter (LVEDD)>73mm (HR: 3.70, p=0.001), exercise periodic breathing (EPB) on cardiopulmonary exercise testing (HR: 2.67, p=0.01), and non-sustained ventricular tachycardia (NSVT)>8 beats on Holter monitoring (HR: 3.47, p=0.027). Predictors of all-cause death were galectin-3>22.5 ng/mL (HR: 3.69, p=0.001), LVEDD>73mm (HR: 3.35, p=0.003), EPB (HR: 3.06, p=0.006), and NSVT>8 beats (HR: 3.95, p=0.007). The absence of all risk predictors was associated with a 91.1% negative predictive value for the primary outcome and 96.6% for total mortality.

**Conclusions:**

In non-ischemic HF patients, elevated galectin-3 levels did not predict major arrhythmic events but were associated with total mortality. Absence of risk predictors revealed a prevalent subgroup of HF patients with an excellent prognosis.

## Introduction

Despite impressive therapeutic advances, heart failure (HF) is associated with persistently elevated death rates,^1^ and approximately 30% of the overall mortality in HF patients is attributed to sudden cardiac death (SCD).^2^^,^^3^ Implantable cardioverter defibrillators (ICD) are an established treatment strategy to prevent SCD, particularly in HF due to coronary artery disease, as several clinical trials have demonstrated their unquestionable beneficial effects in clinical outcomes.^4^^–^^6^ However, there is an ongoing debate on the overall efficacy of ICD implantation in patients with non-ischemic cardiomyopathy (NICM). Clinical studies addressing this specific scenario had conflicting results. In the DEFINITE and DANISH trials, which enrolled only NICM patients, all-cause mortality reduction was not achieved, although SCD decrease was detected.^7^^,^^8^

This heterogeneity in efficacy can be partly explained by the disparity in SCD risk for different HF etiologies. Ischemic heart disease is notably associated with an increased risk of life-threatening arrhythmias, attributable mostly to the presence of fibrotic scars involved in arrhythmogenesis.^6^ Myocardial fibrosis, nonetheless, is ubiquitous in HF, irrespective of etiology, and has been consistently associated with risk of SCD.^9^^–^^12^ Late gadolinium enhancement (LGE) on cardiac magnetic resonance (CMR) imaging is a non-invasive diagnostic tool that identifies myocardial fibrosis and has been proposed as a potential predictor of arrhythmic events and mortality in NICM.^12^^,^^13^ Clinical use of LGE-CMR is limited because of its costs, contraindications in common HF scenarios, and restricted availability worldwide. A simple serum biomarker capable of identifying the burden of myocardial fibrosis could potentially help stratify the risk of SCD, with broader application.^14^^,^^15^ Galectin-3 has been recently evaluated as a biomarker of cardiac remodeling and fibrosis, as its production is directly involved in the initiation and progression of tissue scarring.^15^ Preliminary studies have demonstrated that high serum galectin-3 levels predict sustained ventricular arrhythmias in HF patients at high risk for SCD and major cardiac events in dilated and hypertrophic cardiomyopathy.^10^^,^^16^

The present study aimed to assess whether levels of galectin-3 predict arrhythmic events and total mortality in a NICM patient cohort, thus adding predictive value beyond other known risk markers.^17^

## Methods

We performed a prospective observational study that enrolled adult patients under optimized HF treatment in a dedicated HF outpatient clinic at Hospital de Clínicas de Porto Alegre (Porto Alegre, RS, Brazil) from March 2011 to November 2017. Participants had a previous diagnosis of systolic HF of non-ischemic etiology. Systolic HF was defined as LVEF<40%. Although the inclusion criteria allowed assessing LVEF by either two-dimensional transthoracic echocardiography or CMR, all patients participating in the current protocol were initially evaluated by echocardiography, preferably by Simpson’s biplane method. Non-ischemic etiology was defined as the absence of atherosclerotic coronary lesions>75% on coronary angiography or absence of necrotic or ischemic areas on cardiac single-photon emission computed tomography (SPECT) or LGE-CMR. Exclusion criteria were history of SCD, previous cardiogenic syncope, previous sustained ventricular tachycardia (VT), advanced cerebrovascular disease, or life expectancy lower than one year due to non-cardiovascular diseases. The study protocol was approved by the Ethics Committee of Hospital de Clínicas de Porto Alegre, and written informed consent was obtained from all participants. Study protocol procedures involved a detailed clinical evaluation, routine laboratory tests, including B-type natriuretic peptide (BNP) or N-terminal pro-B-type natriuretic peptide (NT-proBNP), non-invasive cardiac exams, and invasive electrophysiological study (EPS). Non-invasive cardiac exams consisted of routine resting electrocardiogram (EKG), 24 h continuous EKG recording (Holter), and cardiopulmonary exercise testing (CPET). During invasive EPS, blood was drawn for galectin-3 analysis.

### EKG and 24 h Holter monitoring

Twelve-lead EKG was performed with a digital device (Mortara ELI 350, Mortara Instrument, Milwaukee, Wisconsin, USA), and 24 h Holter monitoring was carried out using a digital record (SEER Light) and analyzed with the GE Marquette MARS (GE Healthcare, Wauwatosa, Wisconsin, USA) software by an experienced cardiologist. Non-sustained VT (NSVT) was defined as a run of 3 or more consecutive premature ventricular beats≥100 bpm.

### Cardiopulmonary exercise testing (CPET)

CPET was performed on a standard treadmill (INBRAMED^TM^ KT 10200, Porto Alegre, Brazil) with a calibrated computer-based gas analyzer (Cortex Biophysik Metalyzer 3B Stationary CPX system, M13B2.1, Leipzig, Germany). A ramp-staged protocol was used, starting at 2.4 km/h with 1–2% inclination, followed by progressive speed increments of 0.1–0.12 km/h every 20 s and slope increments of 0.5–1.0% every 60 s, until volitional fatigue was reached. Traditional CPET variables were evaluated: peak oxygen consumption (peak VO_2_), carbon dioxide production (VCO_2_), minute ventilation (VE), respiratory exchange ratio (RER), VE/VCO_2_ slope, and exercise periodic breathing (EPB). EPB was defined based on the following criteria: (1) 3 or more regular oscillations, clearly discernible from inherent data noise, (2) regularity, defined by the standard deviation (SD) of 3 consecutive cycle lengths (time between 2 consecutive nadirs) within 20% of the average, and (3) minimal average amplitude of ventilatory oscillation of 5 L (peak value minus the average of 2 in-between consecutive nadirs).

### Invasive electrophysiological study

Patients were sedated with midazolam and fentanyl and locally anesthetized with lidocaine. A quadripolar diagnostic catheter was introduced via the right femoral vein and positioned under fluoroscopy in the right ventricular apex. The EP-Tracer system (CardioTek^TM^, Maastricht, Netherlands) was used for programmed ventricular stimulation (PVS), using an output pulse amplitude twice the threshold and pulse width of 1 ms. Stimulation protocol consisted of up to 3 extrastimuli (S2/S3/S4) delivered after a 10-beat drive train. Ten-millisecond decrements in the extrastimulus coupling interval were applied after each cycle until either ventricular refractoriness or a 200 ms coupling interval was reached. This process was repeated under 3 different basal cycle lengths (600, 500, and 400 ms). Sustained VT was defined as monomorphic or polymorphic tachycardia with either ≥30s duration or hemodynamic collapse. When no sustained VT was induced, PVS was repeated with up to 2 extrastimuli (S2/S3) after intravenous isoproterenol infusion (1–4 mcg/min). Induction of monomorphic or polymorphic VT or ventricular fibrillation (VF) with triple extrastimuli was considered a true positive finding in the current analysis. In patients with a previously implanted permanent pacemaker, a non-invasive EPS was performed using the pacemaker programmer under the same protocol.

### Serum galectin-3 measurement

A 20 mL blood sample was taken through the venous sheath before the invasive EPS. The blood sample was centrifuged in a dedicated research laboratory and stored under -70ºC. Galectin-3 was measured in duplicate using an ELISA assay (BG Medicine, Waltham, USA).

### Natriuretic peptides measurement

In the HF outpatient clinic where the patients were followed up, both BNP and NT-proBNP levels were available during the study period. For the current analysis, we defined patients with elevated natriuretic peptide levels as those in the highest quartile of any of them.

### Follow-up and outcomes

Patients had outpatient visits at 3, 6, 18, 24, 30, and 36 months. Those who did not attend follow-up visits were contacted by telephone or received home visits. ICD implantation was decided by the clinical cardiology team involved in routine care, with no interference from the researchers. The primary outcome of the protocol consisted of arrhythmic events (SCD, sustained VT, cardiac syncope, or appropriate ICD therapy). ICD shocks were considered appropriate if caused by VT or VF. There was no ICD standardized programming protocol for tachycardia therapy, but the VF zone was typically set to >200 bpm with at least 1 train of antitachycardia pacing (ATP) prior to shock, while the VT zone was typically set to >180 bpm with at least 3 trains of ATP prior to shock. The secondary outcome of the study was all-cause death. Outcomes were adjudicated by two independent researchers blinded for the baseline assessment. Discordant cases were defined by consensus.

### Statistical analyses

Data are expressed as mean±SD or median and interquartile range (IQR) for continuous variables according to data normality, or as absolute numbers and percentage for categorical variables. Continuous data were added to the regression model and categorized based on quartiles of distribution into 2 groups: patients with values below the 75^th^ percentile (<quartile 3[Q_3_]) and patients with values above or equal to the 75^th^ percentile (≥ Q_3_). The Shapiro-Wilk test was used to determine the normality of all continuous variables. Groups were compared using the unpaired Student’s *t*-test for continuous variables and the chi-square test or Fischer’s exact test for categorical variables. Non-normally distributed variables were compared by the Mann-Whitney U test. For the CPET missing values (n=13), data were imputed according to five multivariate models, built from variables capable of predicting EPB, based on previously validated imputing strategies from Rubin,^18^ which provide input values without losing data accuracy. The survival rate and survival free from serious arrhythmic events in the two groups were determined by the Kaplan-Meier method, and the difference between them was analyzed using the log-rank test. Cox regression was adopted for univariate and multivariate analyses of potential predictors of primary and secondary outcomes. A two-tailed p-value of 0.05 or less was considered statistically significant. Based on a 26% incidence of ICD therapies from a cohort study comprising NICM and ischemic HF, a sample size of 142 patients was calculated (80% statistical power and two-tailed p-value<0.05). All analyses were performed using the SPSS statistical software (version 19; Chicago, USA).

## Results

### Patient characteristics

We enrolled 148 of the 296 HF outpatients screened. Most were male (59.5%), with a mean age of 54.8±13 years. HF etiology was idiopathic cardiomyopathy in 45.4% of cases, hypertensive cardiomyopathy in 16.9%, and alcoholic cardiomyopathy in 12.2%. Most subjects were classified in New York Heart Association (NYHA) functional class I or II (42.6% and 39.9%, respectively), and the mean LVEF was 27.4±7.5%. Pharmacological treatment was optimized in the majority of patients: 97% were on a beta-blocker and either an angiotensin-converting enzyme inhibitor or an angiotensin receptor blocker, 70% were taking spironolactone, and 81% were using digoxin. Table 1 describes clinical characteristics of the whole cohort stratified by galectin-3 levels.

**Table 1:** Clinical characteristics according to galectin-3 levels

		All patients (n=148)	Upper quartile GAL-3>22.5 ng/mL (n=36)	Lower quartiles GAL-3≤22.5 ng/mL (n=112)	p-value
Age (years)		54.8±12.7	63±9.3	52.2±12.6	<0.001
Male gender (%)		88 (59.5)	20 (55.6)	68 (61.3)	0.54
**NYHA class (%)**					
	I	63 (42.6)	11 (30.6)	51 (46)	0.12
	II	59 (39.9)	15 (41.7)	44 (40)	
	III	26 (17.6)	10 (28.8)	16 (14.4)	
	IV	0	0 (0)	0 (0)	
**Etiology (%)**					
	Idiopathic	67 (45.3)	13 (36.1)	54 (48.6)	0.38
	Hypertensive	25 (16.9)	7 (19.4)	18 (16.2)	
	Alcoholic	18 (12.2)	4 (11.1)	14 (12.6)	
	Chagas disease	7 (4.7)	3 (8.3)	4 (3.6)	
	Valvular	4 (2.7)	2 (5.6)	2 (1.8)	
	Other	27 (18.2)	7 (19.5)	19 (17.1)	
**Physical examination**					
SBP (mmHg)		119.3±21.6	122.6±22.5	118.1±21.3	0.28
DBP (mmHg)		74.7±12.7	74.6±13	74.8±12.8	0.95
**Laboratory tests**					
Hemoglobin (g/dL)		13.4±1.6	12.6±1.9	13.6±1.4	0.008
Lymphocytes (/mm³)		2099.2±848	1825±787	2193±852	0.02
Creatinine (mg/dL)		1.1±0.73	1.6±1.1	1.0±0.4	0.002
Sodium (mEq/L)		140±2.8	140±2.9	140±2.8	0.86
Potassium (mEq/L)		4.6±0.4	4.6±0.4	4.6±0.3	0.82
Uric acid (mg/dL)		7.5±2.2	8.6±2.4	7.1±2.1	<0.001
Glucose (mg/dL)		118±49.6	117.5±40.3	118.4±52.6	0.92
Total cholesterol (mg/dL)		180.6±42.6	188.4±42.6	177.3±41	0.16
LDL (mg/dL)		104.7±37.2	110.5±37.6	102.3±36.8	0.26
Galectin-3 (ng/mL)		19±9.4	31.6±10.7	14.9±10.7	<0.001
BNP (pg/mL)		116.4 (59.7–295)	158 (77–289)	106.6 (53–298)	0.30
NT-proBNP (pg/mL)		1145 (392–2590)	4776 (1549–15852)	741 (314–2291)	0.005
**Echocardiography**					
	LVEF (%)	27.4±7.5	27.3±7.6	27.4±7.5	0.97
	Left atrium (mm)	47.3±6.6	48.7±7	46.9±6.4	0.15
	LVEDD (mm)	67.5±10.2	65.3±7	68.2±11	0.13
	LVESD (mm)	58.7±10.1	56.6±8.5	59.4±10.6	0.15
**EKG**					
Atrial fibrillation		22 (14.9)	8 (22)	14 (12.6)	0.16
LBBB		60 (40.8)	18 (50)	42 (37.8)	0.46
**24-hour Holter monitoring**					
NSVT (%)		54 (36.5)	12 (33.3)	42 (38.5)	0.57
NSVT>8 beats		11 (7.4)	1 (8.3)	9 (21.4)	0.54
**Cardiopulmonary exercise testing**					
Peak VO_2_ (mL/kg/min)		18±5.1	14.7±4.3	19±4.9	<0.001
VE/VCO_2_ slope		41.5±11.7	44.3±12.5	40.8±11.5	0.14
EPB (%)		26 (17.5)	3 (8.3)	23 (20.5)	0.14
**EPS (%)**					
No induction		129 (87.2)	34 (94.4)	94 (84.7)	0.10
SMVT		10 (6.8)	0	10 (9)	
SPVT		5 (3.4)	1 (2.8)	4 (3.6)	
Ventricular fibrillation		3 (2)	0	3 (2.7)	
HV interval (ms)		52.6±10.4	54.2±11.3	52.2±10.1	0.34
**Medication**					
Beta-blocker (%)		144 (97.3)	36 (100)	107 (96.4)	0.57
ACEi or ARB (%)		144 (97.3)	32 (88.9)	111 (99.1)	0.003
Spironolactone (%)		103 (69.6)	21 (58.3)	82 (73.9)	0.07
Digoxin (%)		121 (81.8)	31 (86.1)	90 (81.1)	0.49
Antiarrhythmic drug (%)		8 (5.4)	1 (2.8)	7 (6.3)	0.41

Data expressed as mean±standard deviation, median (Q1–Q3), or absolute numbers (percentage). GAL-3: galectin-3; NYHA: New York Heart Association; SBP: systolic blood pressure; DBP: diastolic blood pressure; LDL: low-density lipoprotein; BNP: B-type natriuretic peptide; NT-proBNP: N-terminal pro-B-type natriuretic peptide; LVEF: left ventricular ejection fraction; LVEDD: left ventricular end-diastolic diameter; LVESD: left ventricular end-systolic diameter; EKG: electrocardiography; NSVT: non-sustained ventricular tachycardia; VO2: oxygen consumption; VE/VCO2 slope: ventilatory efficiency; EPB: exercise periodic breathing; EPS: electrophysiological study; SMVT: sustained monomorphic ventricular tachycardia; SPVT: sustained polymorphic ventricular tachycardia; ACEi: angiotensin-converting enzyme inhibitor; ARB: angiotensin II receptor blocker; LBBB: left bundle branch block.

### Follow-up and outcomes

Median follow-up was 941 days (IQR: 440–1241 days). All patients had at least two follow-up visits, and only three could not be reached. The primary outcome (Table 2) occurred in 26 patients (17.5%): SCD in 13 (8.7%), cardiac syncope in 5 (3.3%), appropriate ICD therapy in 7 (4.7%), and sustained VT in 1 (0.7%). During follow-up, 30 (20.2%) patients died, and 10 of these deaths were adjudicated as cardiovascular death (6.7%). Eight patients (5.4%) underwent heart transplant. There were 81 hospital admissions for decompensated HF. Forty-eight patients underwent device implant: 17 ICDs with cardiac resynchronization therapy (CRT-D), 19 single-chamber ICDs, 5 cardiac resynchronization therapy with pacemaker (CRT-P), 4 dual-chamber ICDs, and 4 single-chamber pacemakers.

**Table 2:** Univariate analysis and Cox proportional hazard model for the primary outcome (major arrhythmic events)

	Univariate analysis			Multivariate analysis		
	HR	95%CI	p	HR	95%CI	p
Galectin-3 (for each 1 ng/mL)	1.003	0.97–1.04	0.877			
Galectin-3>22.5 ng/mL	1.13	0.47–2.70	0.787			
Atrial fibrillation	1.86	0.75–4.60	0.182			
LVEF<20%	0.69	0.21–2.30	0.691			
LVEDD>73 mm	4.13	1.91–8.90	<0.001	3.70	1.69–8.1	0.001
Peak VO_2_<14.2 mL/kg/min	1.69	0.75–3.90	0.203			
VE/VCO_2_ slope>48.4	2.32	1.05–5.10	0.037			
EPB	3.37	1.52–7.40	0.030	2.67	1.19–6.0	0.017
HV interval>59 ms	2.23	1.01–4.90	0.047			
NSVT>8 beats	3.27	1.11–9.70	0.030	3.47	1.15–10.5	0.027
Positive EPS	1.58	0.54–4.60	0.403			
Elevated natriuretic peptides	2.75	1.26–6.01	0.011			

HR: hazard ratio; LVEF: left ventricular ejection fraction; LVEDD: left ventricular end-diastolic diameter; peak VO_2_: peak oxygen consumption; NSVT: non-sustained ventricular tachycardia; EPB: exercise periodic breathing; EPS: invasive electrophysiological study.

### Galectin-3 levels

Mean galectin-3 level was 19±9.4 ng/mL, and the median was 16 ng/mL (IQR: 13.1–22.5). Clinical data stratified by the upper quartile of galectin-3 levels (>22.5 ng/mL) are shown in Table 1. Galectin-3 levels did not differ in patients with or without the primary outcome, and quartiles of galectin-3 (>22.5 ng/mL) also did not differentiate patients with serious arrhythmic events (7 [19.4%] patients in the upper quartile versus 19 [17%] patients in lower quartiles; p=0.73). However, galectin-3 levels stratified by quartiles were significantly different for overall mortality (13 [36.1%] patients in the upper quartile versus 17 [15.2%] patients in lower quartiles; p=0.007) and for HF hospitalization (50 [34%] patients in the upper quartile versus 31 [21%] patients in lower quartiles; p<0.001) during follow-up.

### Univariate and multivariate analyses

In univariate analyses, significant predictors of major arrhythmic events (Table 2) were the highest quartile of left ventricular end-diastolic diameter (LVEDD) on echocardiography (hazard-ratio — HR: 4.13, p<0.001), the highest quartile of the VE/VCO_2_ slope (HR: 2.32, p=0.03), the presence of EPB on CPET (HR: 3.37, p=0.03), the highest quartile of the HV interval on invasive EPS (HR: 2.23, p=0.04), and NSVT>8 beats on Holter monitoring (HR: 3.27, p=0.03). In this analysis, galectin-3 levels, both continuous and stratified by quartiles, were not significant predictors of the primary outcome (HR: 1.13, p=0.78). In the multivariate Cox regression model, variables that remained significantly associated with serious arrhythmic events were LVEDD (HR: 3.70, p=0.001), presence of EPB (HR: 2.67, p=0.01), and NSVT>8 beats (HR: 3.47, p=0.027). Similar results were obtained using LVEDD indexed to body surface area (LVEDD>40 mm/m^2^ representing the 75^th^ percentile; HR: 3.34; 95% confidence interval 1.50–7.45; p=0.003).

Predictors of total mortality in univariate analyses were similar, but also included the highest quartile of galectin-3 levels (HR: 2.20, p=0.03), peak VO_2_ (HR: 0.92, p=0.04), and the highest quartile of the HV interval (HR: 2.80, p=0.005). Independent predictors of total mortality in the multivariate model (Table 3) were the highest quartile of galectin-3 (HR: 3.69, p=0.001), the highest quartile of LVEDD (HR: 3.35, p=0.003), the presence of EPB (HR: 3.06, p=0.006), and NSVT>8 beats (HR: 3.95, p=0.007). Similar results were obtained using LVEDD indexed to body surface area (LVEDD>40 mm/m^2^ representing the 75^th^ percentile; HR: 3.77; 95% confidence interval 1.77–8.02; p=0.001).

**Table 3:** Univariate analysis and Cox proportional hazard model for the secondary endpoint (total mortality)

	Univariate analysis			Multivariate analysis		
	HR	95%CI	p	HR	95%CI	p
Galectin-3 (for each 1 ng/mL)	1.024	0.99–1.05	0.057			
Galectin-3>22.5 ng/mL	2.20	1.07–4.50	0.033	3.69	1.7–8.19	0.001
Atrial fibrillation	0.98	0.34–2.80	0.980			
LVEF (for each 1%)	0.96	0.91–1.01	0.098			
LVEF<20%	1.40	0.57–3.40	0.461			
LVEDD>73 mm	3.02	1.44–6.30	0.003	3.35	1.53–7.34	0.003
Peak VO_2_ (mL/kg/min)	0.92	0.86–0.99	0.042			
Peak VO_2_<14.2 mL/kg/min	1.20	0.53–2.70	0.656			
VE/VCO_2_ slope>48.4	1.92	0.89–4.10	0.092			
EPB	2.91	1.36–6.20	0.006	3.06	1.38–6.77	0.006
HV interval>59 ms	2.80	1.36–5.80	0.005	1.98	0.95–4.13	0.068
NSVT>8 beats	3.31	1.26–8.70	0.015	3.95	1.45–10.73	0.007
Positive EPS	0.95	0.29–3.10	0.993			
Elevated natriuretic peptides	3.44	1.67–7.06	0.001			

HR: hazard ratio; LVEF: left ventricular ejection fraction; LVEDD: left ventricular end-diastolic diameter; peak VO_2_: peak oxygen consumption; EPB: exercise periodic breathing; NSVT: non-sustained ventricular tachycardia; EPS: invasive electrophysiological study.

### Predictive values

Positive predictive values (PPV) for the primary outcome were low for individual parameters (Table 4); the only variable associated with the highest PPV was EPB (38.4%), while the one with the greatest negative predictive value (NPV) was LVEDD>73mm (88.3%). Ninety patients (61% of the study sample) presented none of the 3 variables independently associated with the primary outcome, leading to an NPV of 91.1%. Similar findings were observed for total mortality: NSVT>8 beats was the predictor associated with the highest PPV (45.5%), while HV interval>59 ms showed the highest NPV (84.9%). Patients without any of the 5 variables independently associated with risk had an NPV of 96.3% for all-cause death.

**Table 4:** Positive and negative predictive values according to risk factors

Primary outcome (major arrhythmic events)				
	All patients (n=148)	With event (n=26)	PPV% (95%CI)	NPV% (95%CI)
LVEDD>73 mm	36	13	36.1 (24.9–49)	88.3 (83.7–91.8)
EPB	26	10	38.4 (24.2–54.9)	86.8 (82.9–90)
NSVT>8 beats	11	4	36.3 (15.2–64.4)	83.7 (81.2–85.9)
Absence of all three	90	8		91.1 (85.4–94.8)
**Secondary outcome (total mortality)**				
	**All patients (n=148)**	**With event (n=30)**	**PPV% (95%CI)**	**NPV% (95%CI)**
GAL-3>22.5 ng/mL	36	13	36.1 (24.6–49.4)	84.8 (80.1–88.5)
LVEDD>73 mm	36	12	33.3 (22.1–46.8)	83.9 (79.3–87.6)
EPB	26	10	38.4 (24.3–55.2)	83.6 (79.6–86.9)
NSVT>8 beats	11	5	45.5 (21.4–71.8)	81.4 (78.8–83.8)
HV interval>59 ms	35	13	37.1 (25.3–50.7)	84.9 (80.3–88.6)
Absence of all five	55	2		96.3 (85.2–99.0)

GAL-3: galectin-3; LVEDD: left ventricle end-diastolic diameter; EPB: exercise periodic breathing; NSVT: non-sustained ventricular tachycardia.

Figure 1 demonstrates the survival curves for different levels of galectin-3, adjusted for the other predictors of all-cause mortality in the Cox regression model (p<0.001). Figure 2 shows the Kaplan-Meier survival curve for the primary outcome stratified by the number of predictor variables (LVEDD, EPB, NSVT>8 beats; log-rank p value<0.001). Figure 3 depicts the Kaplan-Meier survival curve for all-cause mortality stratified by the number of risk markers (galectin-3>22.5 ng/mL, LVEDD>73mm, EPB, NSVT>8 beats, HV interval>59 ms). HF patients with more than 3 risk factors had an ominous prognosis, with a mortality rate>80% after 3 years of follow-up.

**Figure 1:** Kaplan-Meier survival curve for total mortality according to levels of serum galectin-3.

**Figure 2:** Kaplan-Meier survival curve for the primary outcome (serious arrhythmic events) stratified by the number of risk factors (dilated left ventricular end-diastolic diameter, exercise periodic breathing on cardiopulmonary exercise testing, and non-sustained ventricular tachycardia on Holter monitoring).

**Figure 3:** Kaplan-Meier curve for the secondary outcome (total mortality) stratified by the number of risk factors (elevated levels of galectin-3, dilated left ventricular end-diastolic diameter, exercise periodic breathing on cardiopulmonary exercise testing, non-sustained ventricular tachycardia on Holter monitoring, and increased HV interval).

## Discussion

In this prospective cohort of NICM HF patients, galectin-3 levels were not an independent predictor of major arrhythmic events (SCD, cardiac syncope, sustained VT, or ICD appropriate therapy). However, higher galectin-3 levels were independently associated with overall mortality. Previously, we had identified 3 clinical predictors of arrhythmic events (LVEDD, EPB, and NSVT on 24-hour Holter monitoring), which were confirmed by the current analysis.^17^ In a clinical scenario in which ICD implants are under scrutiny, our data may help select which patients would most benefit from an invasive and costly therapy. Serum levels of galectin-3 may be used to further stratify risk and help in prognostication.^18^

Galectins are a large family of lectins that bind to ß-galactosides. Primarily located in the cytoplasm, they can also be found in the nucleus or the extracellular matrix. Extracellular galectin-3 exhibits numerous autocrine and paracrine effects, including cell adhesion, activation and chemoattraction of certain cell types, mainly those related to the extracellular matrix. Galectin-3 affects various biological processes, such as cellular homeostasis, immune responses, organogenesis, and angiogenesis.^19^ Henderson et al. reported that disruption of the galectin-3 gene blocked hepatic stellate cell activation and collagen expression in the liver, attenuating hepatic fibrosis.^20^ Aldosterone led to galectin-3 expression in the aortic tunica media in animal models and, in turn, its overexpression increased type I collagen production.^21^

The role of galectin-3 as a predictor of future clinical events has been partially assessed in several cardiovascular scenarios. Higher galectin-3 levels were related to new-onset atrial fibrillation (AF) 3–5 days after ST-segment elevation myocardial infarction.^22^ Recent reports have also indicated the association of galectin-3 levels with paroxysmal AF^23^ and persistent AF.^24^ Concentrations of sST2 and galectin-3 were significantly higher in hypertrophic cardiomyopathy patients than in controls, but neither marker had a significant relationship with SCD risk, history of syncope, or family history of SCD.^25^

The predictive role of galectin-3 has also been assessed in studies of mixed HF etiologies, but few have explored its role as a predictor of arrhythmic events specifically in non-ischemic HF patients. Francia et al. investigated 75 HF patients who underwent ICD implantation and found that galectin-3 levels were higher in those with VR or VF. Most of their sample (60%), however, had an ischemic etiology.^16^ Hu et al.^10^ assessed both galectin-3 levels and LGE on CMR in a prospective cohort that enrolled patients with NICM (46% with dilated cardiomyopathy and 56% with hypertrophic cardiomyopathy). Both galectin-3 levels and the presence of LGE were independent predictors of major cardiac events. No specific analysis related to arrhythmic risk or total mortality was performed.^10^ Recently, Competence Network Heart Failure investigators evaluated the role of sST2 and galectin-3 in patients with non-ischemic dilated cardiomyopathy and obtained intriguing results. While sST2 was associated with cardiac and overall mortality, galectin-3 levels had no impact on the risk of future events as a continuous variable, but the intermediate tertile of galectin-3 was significantly associated with a better prognosis.^26^ Nonetheless, our findings suggest that elevated galectin-3 levels might not predict arrhythmic risk in a selected sample of non-ischemic HF patients but could be an important marker of overall mortality. These results are in agreement with a meta-analysis of 9 studies that included a heterogeneous group of HF patients and reported that, for every 1 ng/mL of galectin-3 elevation, the mortality rate increased by 28%.^27^

In the current analysis, the absence of all independent predictors of risk for arrhythmic events (Figure 2) or total mortality (Figure 3) revealed a subgroup of patients with excellent prognosis in a follow-up of up to four years. These results strengthen our previous findings^17^ and point out that better risk stratification is feasible in non-ischemic HF patients.

Some methodological aspects of our protocol deserve consideration. We lost contact with only three patients, so they were censored in the last visit. Asymptomatic ventricular arrhythmias could not be detected, as only 48 patients of our cohort underwent device implantation. LGE-CMR has been suggested as a valid tool for prognosis stratification of HF, but only a few of our patients underwent CMR, precluding its analysis as a prognostic factor in this cohort. CMR, however, is not widely available to most HF patients worldwide. We acknowledge that our sample size and number of events are relatively small, and as such, our results should be considered hypothesis-generating. Still, most previous studies had similar or smaller sample sizes. Finally, our findings deserve future prospective validation before broad clinical applicability can be proposed.

## Conclusion

In this prospective cohort of non-ischemic HF patients on optimized medical treatment, galectin-3 levels did not predict major arrhythmic events. Three variables were confirmed as risk markers for the primary outcome: LVEDD>73 mm, EPB on CPET, and NSVT>8 beats on Holter monitoring. Elevated galectin-3 levels were independently associated with total mortality. Absence of all risk predictors revealed a substantially prevalent subgroup of patients with an excellent prognosis. In the current scenario of uncertainty about ICD advantages in NICM HF patients, our results might help establish future strategies to identify the patients who would most benefit from ICDs.
